# Myocardial infarction in patients with severe beta thalassaemia: a case series

**DOI:** 10.1186/s12245-023-00495-z

**Published:** 2023-03-09

**Authors:** Anuja Premawardhena, Shamila De Silva, Megha Rajapaksha, Vishaka Ratnamalala, Jemimah Nallarajah, Gamini Galappatthy

**Affiliations:** 1 Department of Medicine: Faculty of Medicine, University of Kelaniya, Kelaniya, Sri Lanka; 2 Adolescent & Adult Thalassaemia Unit, North Colombo (Teaching) Hospital, Ragama, Sri Lanka; 3 Department of Haematology, National Hospital of Sri Lanka, Colombo, Sri Lanka; 4 Institute of Cardiology, National Hospital of Sri Lanka, Colombo, Sri Lanka

## Abstract

**Background:**

Cardiac disease remains a dominant if not the most important cause of morbidity and mortality in patients with thalassaemia, particularly in those with thalassaemia major. Myocardial infarction and coronary artery disease however are rarely reported.

**Case presentations:**

Three older patients with three distinct thalassaemia syndromes presented with acute coronary syndrome. Two were heavily transfused whilst the other was a minimally transfused patient. Both heavily transfused patients had ST-elevation myocardial infarctions (STEMI) while the minimally transfused patient had unstable angina. Coronary angiogram (CA) was normal in two patients. One patient who developed a STEMI had a 50% plaque. All three were managed as standard ACS, although the aetiology appeared non-atherogenic.

**Conclusions:**

The exact etiology of the presentation, remains a mystery and therefore the rational use of thrombolytic therapy, carrying out angiogram in the primary setting, using and continuing antiplatelet and high dose statins all remains unclear in this sub group of patients.

## Background

Cardiac disease due to iron overload is the commonest cause of death in patients with thalassaemia major, accounting for almost 70% of deaths in some series [1, 2]. Cardiac iron overload related deaths may be less common in patients with non-transfusion dependent thalassaemia (NTDT), including in patients with haemoglobin E-beta thalassaemia where malignancies and infections seem to be the dominant causes for mortality [3–5]. Pulmonary hypertension that develops due to a multitude of reasons is also recognized as an important contributor to cardiac related mortality, particularly in older splenectomised patients with thalassaemia intermedia [6].

Myocardial infarction has only rarely been reported in patients with thalassaemia. There is an observation that those with beta thalassaemia trait may be protected against developing coronary artery disease (CAD), and it is well known that patients with thalassaemia tend to have hypocholesterolemia with low LDL level [7, 8]. However, there is also a suggestion that patients with thalassaemia, especially those with the intermediate form, have a higher chance of developing premature atherosclerosis and vascular abnormalities resembling pseudo- xanthoma elasticum [9, 10]. Many studies have demonstrated a prothrombotic state in patients with thalassaemia [11–13].

The first documented myocardial infarction in a person with thalassaemia was in a patient with thalassaemia major in 2004. The patient was thrombolysed with streptokinase for a ST-elevation myocardial infarction (STEMI) but there were no plaques on a coronary angiogram carried out a month later. The authors suggested that thromboembolism or vasospasm may have been causative [14]. A patient with thalassaemia intermedia reported with a STEMI in 2008 however had evidence of two coronary plaques, one of which caused a 90% obstruction in the left anterior descending (LAD) artery. He underwent primary percutaneous intervention (PCI). [15].

We were unable to find any further reports of acute coronary syndrome (ACS) in persons with thalassemia syndromes in the literature. Currently, there is no clear idea on pathogenesis or the best strategy for management of such persons in the emergency care setting.

It is in this backdrop that we encountered three coronary events over a period of four months in adult patients with thalassaemia receiving treatment at a single center in Sri Lanka.

### Patient 1

A 34-year-old woman with haemoglobin E-beta thalassaemia (IVS1-5(G-C)/Hb E; α-^4.2^/αα) presented to a hospital in Southern Sri Lanka with epigastric pain and severe central chest pain lasting for more than 18 hours. She complained of radiating pain to the neckleft arm pain and difficulty in breathing. She was diagnosed to have an anterior STEMI based on ECGs and was treated with aspirin 300 mg, clopidogrel 300 mg, atorvastatin 40 mg and enoxaparin 40 mg sub-cutaneously on admission. She had not been thrombolysed due to the late presentation. High sensitivity cardiac troponin was 136.6 ng/dL on admission and rose to 1036 ng/dL within six hours. She had an uneventful recovery after a 6-day hospital stay. Results of further investigations are in Table 1.

**Table 1:** Results of investigations during hospital stay for acute coronary event in the three patients

	Patient 1	Patient 2	Patient3
Highest Troponin Titre	1036 (< 12)	normal	2326 (< 12)
White cell count × 10^9^/L	14.5	3.0	6.84
Hemoglobin g/dL	8.4	7.8	9.3
Platelet count × 10^9^/L	337	252	576
CRP mg/dL		4.1	
SGPT IU/L	703	433	114
SGOT IU/L		716	175
Serum creatinine mg/dL	0.4	0.52	0.98

Echocardiogram at discharge showed an ejection fraction of 60%. Coronary angiogram done a week later showed a normal main coronary, a 50% occlusion of the proximal LAD, and mild disease in the mid vessel. The right coronary artery was normal.

The patient was diagnosed with thalassemia at seven years of age, when she presented with a haemoglobin of 6.2 g/dL. She had been managed at three different transfusion centers across the country during different stages of her life, but throughout she had been transfused either monthly or once in 2–3 months. Mean pre-transfusion haemoglobin was 7.8 g/dL.

Splenectomy was performed at the age of 22 years. Mean platelet count post-splenectomy was 550,000/mm^3^. The highest recorded serum ferritin value was 8500 ng/ml in 2016 and the most recent was 1130 ng/ml in May 2022. Cardiac and liver T2^*^MRI had been done once in 2017 which read 9.2 ms and 10.7 ms respectively. She was on hormone replacement therapy for hypogonadism, thyroxine for hypothyroidism, and calcium and vitamin D3 replacement for hypoparathyroidism. She was also on treatment for diabetes mellitus for the past five years with premixed insulin and had variable glycaemic control. Her BMI was 21.8 kg/m^2^. At the time of admission, she was on dual chelators with oral deferasirox 30 mg/kg/day and desferrioxamine 30 mg/kg/day subcutaneously, a regime she was following for the previous six months. Her pre-event echocardiogram was normal with an ejection fraction of 60% and no evidence of pulmonary hypertension.

She was vaccinated against COVID-19 with two doses of the Sinopharm vaccine, with the second dose given two months before the acute coronary event. She developed a mild COVID-19 infection in October 2021 which was managed at home without any complications.

### Patient 02

A 44-year-old woman with beta thalassaemia intermedia diagnosed at 24 years (genotype IVS1-5(G-C))/ β;ααα/ααα] presented to the emergency department with severe bilateral submammary pain lasting for more than one hour, associated sweating, an episode of fainting and vomiting. ECG on admission showed T inversion in LII, LIII, aVf, V3, and V4. Subsequent ECGs showed dynamic changes spreading to V5. Troponin titers on admission and six hours later remained un-elevated. She was treated for unstable angina with four doses of enoxaparin subcutaneously. She received one unit of blood transfusion while in hospital. Investigations showed elevated liver enzymes which gradually reduced over the hospital stay of three days. Results of further investigations are shown in Table 1.

The patient had a mild form of beta thalassaemia intermedia and attended clinic only occasionally. Examination did not show a clinically palpable spleen. She had been transfused only 10 units of blood during her lifetime, with the last transfusion in 2011. Almost all the blood transfusions had been given during her pregnancy. Her mean haemoglobin level was 7.5 g/dL and mean platelet count was 250–300,000/μL. She had no previous history of chest pain, shortness of breath or symptoms suggestive of angina. Serum ferritin level was 644 ng/dL in 2019 at her last clinic visit. She had no major complications of thalassemia of note and had never been on iron chelators.

She had been given two doses of the Sinopharm COVID-19 vaccine, the second dose in July 2021. She has a mild upper respiratory infection in February 2022 but did not test for COVID-19.

### Patient 3

A 21-year-old male patient with beta thalassaemia major presented to the emergency department with severe retrosternal chest pain and shortness of breath of three hours duration. He had associated symptoms of sweating and palpitations. ECG on admission showed striking ST elevations in the inferior and lateral leads. Troponin titer was highly positive at 236 ng/dL. He was thrombolysed with tenecteplase in the emergency treatment unit, followed by subcutaneous enoxaparin. The chest pain reduced but ECG changes did not show resolution and he was transferred for urgent per-cutaneous intervention (PCI). At the coronary care unit, a coronary angiogram showed normal coronary vasculature. Echocardiogram showed global hypokinesia with an ejection fraction < 30%. The patient developed repeated episodes of ventricular tachycardia refractory to treatment and succumbed during one episode.

The patient was on regular transfusions from the time of diagnosis at 6 months of age. Mean pre-transfusion haemoglobin was 9.5 g/dl during the past year but he was not always well transfused. He had a BMI of 17.4 kg/m^2^. He had undergone splenectomy at the age of 9 years and the mean platelet count was 505,000/μL over the past four years. He had hypogonadism and puberty was induced at the age of 13 years. He had no other endocrine abnormalities. The last serum ferritin available was 5080 ng/ml and there was a rising trend. T2* cardiac MRI done in November 2021 was 5/ms confirming severe myocardial iron load. He was on dual chelators (deferasirox and desferrioxamine) but was poorly compliant with chelator use. Pre-event echocardiogram done six months ago was normal with an ejection fraction > 60% and no evidence of pulmonary hypertension.

He had two Sinopharm vaccines against COVID -19 in July and August of 2021, and there was no record of infection with COVID 19 at any point.

## Discussion

We present case histories of three older patients with three distinct thalassemia syndromes followed up at the same thalassaemia unit, who presented with acute coronary syndrome during a four-month period. Acute coronary syndrome is rare in patients with thalassemia, with literature discussing only a few cases. The coronary arterial pathology in the three patients in our series did not show significant atheromatous disease. The female with unstable angina and the 21-year-old man with STEMI who succumbed during the episode had normal coronary arteries. The 34-year-old woman who developed an anterior STEMI had only a 50%-occluding plaque in the left anterior descending (LAD) coronary artery. In a previous report of a STEMI in a patient with thalassemia major from Israel the authors were not able to identify any plaques on coronary angiography [14].

In our series the two patients who developed STEMI were heavily transfused although their genetic make-up was different, with one having beta thalassaemia major and the other having haemoglobin E-beta thalassaemia. The latter individual had a plaque albeit of moderate significance. It is likely that the pathogenesis of the cardiac events was due to thromboembolism. There is sufficient literature to support a pro-coagulant state in thalassaemia syndromes. Both patients had undergone splenectomy at a young age and had moderately elevated platelet counts. DRVVT and KCT were done to exclude acquired thrombophilic conditions but more extensive testing including testing for Factor V Leiden and protein C, protein S and anti-thrombin level were not done due to cost limitations. In the two previous case reports in the literature no significant association with inherited thrombophilc factors had been reported [14, 15].

Based on the 4th Universal Definition of Myocardial Infarction (MI), in a patient if a demonstrable obstruction > 50% is absent in the coronary angiogram but other criteria fulfill a diagnosis of MI the term ‘myocardial infarction with non-obstructive coronary arteries’ (MINOCA) is used, an example of type 2 MI. In the two patients with definite evidence of myocardial infarction, in the absence of significant atheromatous disease a type 2 myocardial infarction could be postulated. Likewise, the aetiology of unstable angina in patient 2 may also be related to a similar pathology. However, the source of the thrombus or embolus if any could not be identified. Coronary arterial vasospasm has been postulated as a probable mechanism for causation of the myocardial infarction in one of the previous cases but no explanations as to why vasospasm should occur in patients in thalassemia could be found in the literature.

We were intrigued by the occurrence of three coronary events over a short period of time in a single unit and wondered if COVID-19 infection could have played a role in the pathogenesis. All three patients had received at least two vaccines for COVID-19 but had contracted mild infections subsequently. There was no evidence of acute COVID infection in any one of them at the time of the events.

Just as much as the aetiology remains unclear how should a similar event be managed in the future? The rational use of thrombolytic and anticoagulants in an acute event remains unclear as is the use of high doses of lipid lowering treatment in the follow-up of these patients. Its perhaps too early to discourage early angiography in thalassaemic patients with suspected ACS because the aetiology remains uncertain.

## Data Availability

Will be made available on request from the authors, including ECHO cardiograms.

## Abbreviations

ACS: Acute coronary syndrome
BMI: Body mass index
CA: Coronary angiogram
CAD: Coronary artery disease
DRVVT: Dilute Russel viper venom test
ECG: Electro cardiogram
KCT: Kaolin clotting time
LAD: Left anterior descending artery
LDL: Low density lipoproteins
MI: Myocardial infarction
MRI: Magnetic resonance imaging
NTDT: Non- transfusion dependent thalassaemia
PCI: Percutaneous coronary intervention
STEMI: ST elevation myocardial infarction

## References

[1] Modell B, Khan M, Darlison M (2008). Improved survival of thalassaemia major in the UK and relation to T2* cardiovascular magnetic resonance. J Cardiovasc Magn Reson.

[2] Arnett DK, Blumenthal RS, Albert MA (2019). 2019 ACC/AHA Guideline on the Primary Prevention of Cardiovascular Disease: A Report of the American College of Cardiology/American Heart Association Task Force on Clinical Practice Guidelines. Circulation.

[3] Musallam KM, Vitrano A, Meloni A, Pollina SA, Karimi M, El-Beshlawy A, Hajipour M, Di Marco V, Ansari SH, Filosa A, Ricchi P, Ceci A, Daar S, Vlachaki E, Singer ST, Naserullah ZA, Pepe A, Scondotto S, Dardanoni G, Bonifazi F, Sankaran VG, Vichinsky E, Taher AT, Maggio A (2021). Survival and causes of death in 2,033 patients with non-transfusion-dependent β-thalassemia. Haematologica.

[4] Vitrano A, Calvaruso G, Lai E, Colletta G, Quota A, Gerardi C, Concetta Rigoli L, Pitrolo L, Cuccia L, Gagliardotto F, Filosa A, Caruso V, Argento C, Campisi S, Rizzo M, Prossomariti L, Fidone C, Fustaneo M, Di Maggio R, Maggio A (2017). The era of comparable life expectancy between thalassaemia major and intermedia: Is it time to revisit the major-intermedia dichotomy?. Br J Haematol.

[5] Premawardhena AP, Ediriweera DS, Sabouhanian A, Allen A, Rees D, de Silva S, et al. Survival and complications in patients with haemoglobin E thalassaemia in Sri Lanka: a prospective, longitudinal cohort study. Lancet Glob Health. 2022;10(1):e134–41. 10.1016/S2214-109X(21)00446-0. Epub 2021 Nov 26. 10.1016/S2214-109X(21)00446-0 PMC8672061 34843671

[6] S, Lamabadusuriya SP, Weatherall DJ, Olivieri NF. Survival and complications in patients with haemoglobin E thalassaemia in Sri Lanka: a prospective, longitudinal cohort study. Lancet Glob Health. 2022 Jan;10(1):e134-e141. doi: 10.1016/S2214-109X(21)00446-0. Epub 2021 Nov 26. PMID: 34843671; PMCID: PMC8672061. 10.1016/S2214-109X(21)00446-0 PMC8672061 34843671

[7] Wood JC (2022). Pulmonary hypertension in thalassemia: a call to action. Blood.

[8] Gallerani M, Scapoli C, Cicognani I, Ricci A, Martinelli L, Cappato R, Manfredini R, Dall'Ara G, Faggioli M, Pareschi PL (1991). Thalassaemia trait and myocardial infarction: low infarction incidence in male subjects confirmed. J Intern Med.

[9] Haghpanah S, Davani M, Samadi B, Ashrafi A, Karimi M (2010). Serum lipid profiles in patients with beta-thalassemia major and intermedia in southern Iran. J Res Med Sci..

[10] Hahalis G, Kalogeropoulos A, Terzis G, Tselepis AD, Kourakli A, Mylona P, Grapsas N, Alexopoulos D (2011). Premature Atherosclerosis in Non-Transfusion-Dependent β-Thalassemia Intermedia. Cardiology.

[11] Farmakis D, Moyssakis I, Perakis A, Rombos Y, Deftereos S, Giakoumis A, Polymeropoulos E, Aessopos A (2003). Unstable angina associated with coronary arterial calcification in a thalassemia intermedia patient with a pseudoxanthoma elasticum-like syndrome. Eur J Haematol.

[12] Sirachainan N (2013). Thalassemia and the hypercoagulable state. Thromb Res.

[13] Eldor A, Rachmilewitz EA (2002). The hypercoagulable state in thalassemia. Blood.

[14] Fridlender ZG, Rund D (2004). Myocardial infarction in a patient with beta-thalassemia major: first report. Am J Hematol.

[15] El Rassi FA, Ismaeel HA, Koussa SC, Taher AT (2008). Myocardial Infarction in a 28-Year-Old Thalassemia Intermedia Patient. Clin Appl Thromb Hemost.

